# Is the Swallow Tail Sign a Useful Imaging Biomarker in Clinical Neurology? A Systematic Review

**DOI:** 10.1002/mdc3.14304

**Published:** 2024-12-17

**Authors:** Vasilis‐Spyridon Tseriotis, Kyriaki Eleftheriadou, Theodoros Mavridis, Georgios Konstantis, Bjoern Falkenburger, Marianthi Arnaoutoglou

**Affiliations:** ^1^ Department of Neurology Agios Pavlos General Hospital of Thessaloniki Thessaloniki Greece; ^2^ Laboratory of Clinical Pharmacology Aristotle University of Thessaloniki Thessaloniki Greece; ^3^ Department of Neurology Tallaght University Hospital (TUH)/The Adelaide and Meath Hospital, Dublin, Incorporating the National Children's Hospital (AMNCH) Dublin Ireland; ^4^ Department of Neurology, University Hospital and Faculty of Medicine Carl Gustav Carus Technische Universität Dresden Dresden Germany; ^5^ 1st Department of Neurology University Hospital AHEPA, Faculty of Medicine Aristotle University of Thessaloniki Thessaloniki Greece

**Keywords:** dorsolateral nigral hyperintensity, swallow tail sign, diagnosis, biomarker

## Abstract

**Background:**

Loss of dorsolateral nigral hyperintensity (DNH) in iron‐sensitive sequences of Magnetic Resonance Imaging (MRI), also described as “swallow tail sign” (STS) loss, has shown promising diagnostic value in Parkinson's Disease (PD) and Atypical Parkinsonian Syndromes (APS).

**Objective:**

To conduct a bibliometric analysis on substantia nigra MRI and a systematic review on the clinical utility of STS visual assessment on Susceptibility‐Weighted Imaging in various clinical entities.

**Methods:**

VOSviewer's keyword co‐occurrence network was employed using Web of Science (WOS). Complying with the PRISMA statement, we searched MEDLINE, WOS, SCOPUS, ProQuest and Google Scholar for peer‐reviewed studies conducted in vivo, excluding quantitative imaging techniques.

**Results:**

DNH is a relatively novel parameter in substantia nigra MRI literature. Our SWI‐focused review included 42 studies (3281 patients). Diagnostic accuracy of STS loss for PD/APS differentiation from controls and for Lewy Body Dementia differentiation from other dementias was 47.8–98.5% and 76–90%, respectively, with poorer capacity, however, in delineating PD from APS. STS evaluation in idiopathic REM sleep behavior disorder, a sign of prodromal PD, was typically concordant with nuclear scans, identifying subjects with high conversion risk. Iron deposition can affect STS in Multiple Sclerosis and STS loss in Amyotrophic Lateral Sclerosis is linked with multisystem degeneration, with poorer prognosis. In healthy individuals iron‐induced microvessel changes are suspected for false positive results.

**Conclusion:**

STS assessment exhibits potential in different settings, with a possibly intermediate role in the diagnostic work‐up of various conditions. Its clinical utility should be explored further, through standardized MRI protocols on larger cohorts.

Technological advancements in magnetic resonance imaging (MRI) and particularly the implementation of susceptibility‐weighted imaging (SWI) and iron‐sensitive techniques have enabled the distinction of the substantia nigra (SN) from surrounding tissues in the midbrain.^1^ Nigrosome‐1, the largest of the dopaminergic cell clusters lying dorsolateral in the substantia nigra pars compacta (SNc),^1^ is the most prominently affected dopaminergic region in Parkinson's disease (PD).^2^ Recent evidence from three‐dimensional histology within vivo/postmortem iron‐sensitive MRI suggests the partial overlapping of nigrosome‐1 with the “swallow tail sign” (STS),^3^ a comma‐shaped hyperintensity in axial midbrain SWI sequences in healthy individuals, also known as dorsolateral nigral hyperintensity (DNH).^1^, ^4^, ^5^ STS loss/absence has been well‐described in individuals with PD.^4^, ^6^ Although the exact neuroanatomic background remains debatable, low signal on iron‐sensitive MRI sequences (eg, SWI, T_2_*) is thought to be related to decreased neuromelanin content and accumulation of free iron, as a result of degeneration of presynaptic dopaminergic neurons,^2^, ^3^ making the detection of STS abnormalities a specific cellular marker.^3^

STS loss in PD seems to exhibit a diagnostic accuracy comparable to that of DaTScan or 6‐[18F] FDOPA PET.^7^, ^8^ Relatively recent meta‐research has highlighted its value in differentiating patients with PD from healthy individuals with a pooled sensitivity and specificity of 94% and 90%, respectively, that rise up to 99% and 92% when a field strength of 7 Tesla (T) is used.^7^ On the contrary, the use of absence of the STS in the context of differentiation of PD from atypical parkinsonian syndromes (APS) is still questionable.^8^

Research regarding STS loss has expanded to various clinical entities and its value is being investigated in complex pathologies, other than parkinsonian syndromes.^9^, ^10^ Moreover, complex imaging modalities like quantitative susceptibility mapping (QSM) and neuromelanin‐sensitive MRI (NM‐MRI) are being employed for a more in‐depth investigation of the appearance of nigrosome‐1.^11^, ^12^ Among iron‐sensitive techniques, SWI is nowadays widely available in MRI scanners in all clinical settings, demonstrating tremendously high sensitivity in detecting paramagnetic and diamagnetic signals, since it can combine magnitude and phase T_2_*‐weighted images.^13^, ^14^

So far, there are studies concerning the use of iron‐sensitive MRI techniques in neurology or neuroimaging and more specifically the use of STS as diagnostic markers of degenerative parkinsonian syndromes.^7^, ^15^ However, there are no systematic reviews regarding the appearance of nigrosome‐1 and STS loss in various neurological disorders. We aim to report recent literature trends regarding MRI of the SN through a large‐scale bibliometric analysis and to explore possible diagnostic and prognostic value of STS loss in various neurological disorders beyond PD through an extensive systematic review of clinical studies. Moreover, we aspire to shed light on the lateralizing value of STS loss and on the agreement between nuclear imaging and MRI.

## Methods

### Bibliometric Analysis

We used VOSviewer,^16^ a software capable of bibliometric mapping and network generation, focusing on keyword co‐occurrences for the visualization of trends in the field of SN MRI. For this purpose, we searched Web of Science (WOS) for articles using the terms “substantia nigra” and “MRI” or “magnetic resonance imaging”. We set the minimum co‐occurrence number for each keyword to 10. Of the 6506 keywords, 302 met the threshold. After the exclusion of general terms, such as “brain”, “disease”, “disorder”, “adult”, “neurons” and “system” 250 keywords remained. We used a network visualization graph to present our results and a table to demonstrate the top 20 keywords based on the number of occurrence.

### Systematic Review

Our systematic review was conducted and reported according to the PRISMA statement for systematic reviews. A PRISMA checklist can be found in the supplementary material (S1).

#### Search Strategy and Information Sources

We performed a search in electronic databases PubMed/MEDLINE, WOS, SCOPUS, ProQuest and Google Scholar covering all papers published from 2010 until the October 26, 2023 using keywords and controlled vocabulary for the terms “swallow tail sign”, “dorsolateral nigral hyperintensity”, “nigrosome‐1”, “susceptibility‐weighted imaging”, “magnetic resonance imaging”. Forward and backward searching, as well as hand searching, were employed. A complete search strategy is presented in our supplementary material (Table S1).

#### Eligibility Criteria

We included studies in adult human subjects with neurological disorders of any kind (diagnosed by application of the respective criteria) or healthy individuals. The studies had to report on the evaluation of STS appearance through visual inspection by radiologists or neuroradiologists on SWI. Eligible study types included case reports, case series, observational case–control studies, cross‐sectional studies, studies of diagnostic accuracy, and randomized clinical studies. We only included peer‐reviewed articles, with their full texts available in English. Since we expanded our search to gray literature sources (ProQuest and Google Scholar), only peer‐reviewed conference abstracts, letters, and editorial comments were considered eligible, and only if authors presented adequate information.

Animal, post‐mortem, or ex‐vivo studies and studies in pediatric population were excluded. The use of quantitative or mapping techniques, neuromelanin‐sensitive MR imaging, as well as diffusion tensor sequences for the diagnostic performance of STS evaluation served as exclusion criteria due to their complexity and limited accessibility in clinical practice, at least for the present time. If eligible studies included STS evaluation on SWI among other endpoints, we isolated and referred to the results that are relevant to our scope, excluding the rest from data synthesis. Non‐peer‐reviewed publications, review articles and preliminary results from theses or dissertations were excluded.

#### Data Management

Systematic Review Accelerator (SRA)^17^ was used for deduplication. Potentially eligible articles were exported into “Rayyan”, a web‐based application for article screening.^17^ Two reviewers (KE, VST) independently screened titles and abstracts and subsequently full texts, remaining blinded to each other's decisions during the process. Conflicts were resolved with the involvement of a third investigator (TM). We imported eligible studies into the reference manager “Mendeley” (Desktop version 1.19.8). The PRISMA flow diagram was constructed using the interactive R‐based online tool developed by Haddaday et al.^18^

#### Data Extraction and Quality Assessment

Data extraction was done in duplicate by two independent investigators (VST, KE), based on predetermined forms, with the involvement of a third reviewer (TM) in case of discrepancies. Extracted data concerned MRI scan characteristics, neurological disorders of interest, disease duration/severity, comparator groups, diagnostic value/findings and intra‐ or inter‐rater agreement. When those were not presented in the primary studies, they were calculated by us from supplementary materials, if available, using the statistical software R (Version 4.1.2). Two independent reviewers (VST, TM) assessed the methodological quality of the included studies using the QUADAS‐2 tool for Diagnostic Accuracy Studies and the JBI Critical Appraisal Checklist for case reports and for prevalence studies. In case of discrepancy a consensus was reached.

#### Data Synthesis

Due to expected heterogeneity in different study designs, a qualitative synthesis of the above‐mentioned variables (without statistical synthesis) was chosen a priori. We grouped eligible studies into categories for summarization and better result interpretation. Thus, we combined mean values (eg, disease duration/severity) when those were separately reported for different patient subgroups of similar pathologies. If diagnostic accuracy measures were not directly given, we calculated sensitivity, specificity and diagnostic accuracy using true negatives, true positives, false negatives and false positives, when available.

## Results

### Bibliometric Analysis

**Figure 1:**
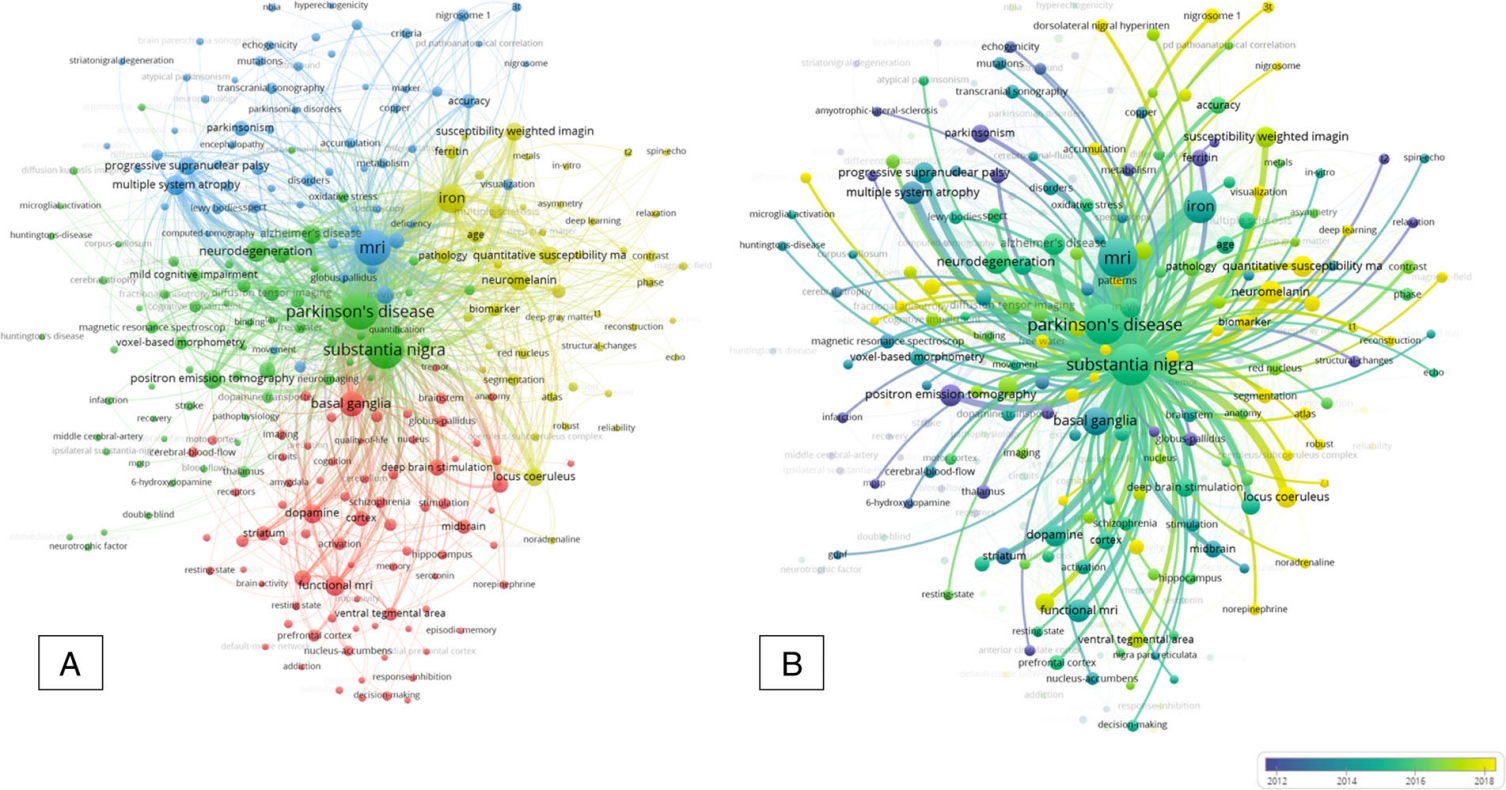
Network visualization graph of keyword co‐occurrence (A) and overlay visualization focusing on the keyword “substantia nigra” (B).

Our large‐scale bibliometric analysis regarding SN MRI is presented in Fig. 1A. In the keyword‐based network visualization graph, the higher weight is depicted with larger circles, representing more occurrences. Different colors represent different clusters. The lines represent co‐occurrences and their thickness varies according to co‐occurrence strength. Additionally, the closer the keywords appear, the more related they are. “Substantia nigra”, “Parkinson's disease” and “MRI” have the highest weight, and thus the highest occurrence. The proximity of the terms “Parkinson's disease” and “substantia nigra” indicates their close relation in the published literature. The terms “MRI”, “diagnosis”, “susceptibility weighted imaging”, “diagnosis”, “iron”, “neuromelanin”, and “quantitative susceptibility mapping” present strong connections and proximity to the aforementioned central terms. Keywords for other neurological entities that occurred in the literature along with the term SN included “multiple system atrophy”, “Alzheimer's disease”, “progressive supranuclear palsy” and “lewy bodies”. The 20 keywords with the highest occurrence rate along with their total link strength, a value that represents the strength of the co‐occurrences, are presented in Table S2.

Moreover, we proceeded to an overlay visualization (Fig. 1B), in which keywords are colored based on the years of their occurrence in the literature. Focusing on all links of the term “substantia nigra”, positron emission tomography comprises the main associated imaging technique in past literature, with a quite strong link. Up until 2016, however, newer techniques were mentioned in the literature in conjunction with SN imaging, such as “voxel‐based morphometry”, “diffusion tensor imaging”, “diffusion kurtosis imaging”, “dopamine transporter”, “SPECT” and “transcranial sonography”. More recently the investigation of “iron” and “neuromelanin” occurred along with the appropriate MRI modalities: “susceptibility weighted imaging”, “neuromelanin‐sensitive MRI” and “quantitative susceptibility mapping”. “Dorsolateral nigral hyperintensity”, “hyperintensity”, “nigrosome” and “nigrosome 1” are specific parameters, recently mentioned in publications relating to MRI and/or specific SWI sequences for visualization of the SN.

### Systematic Review

**Figure 2:**
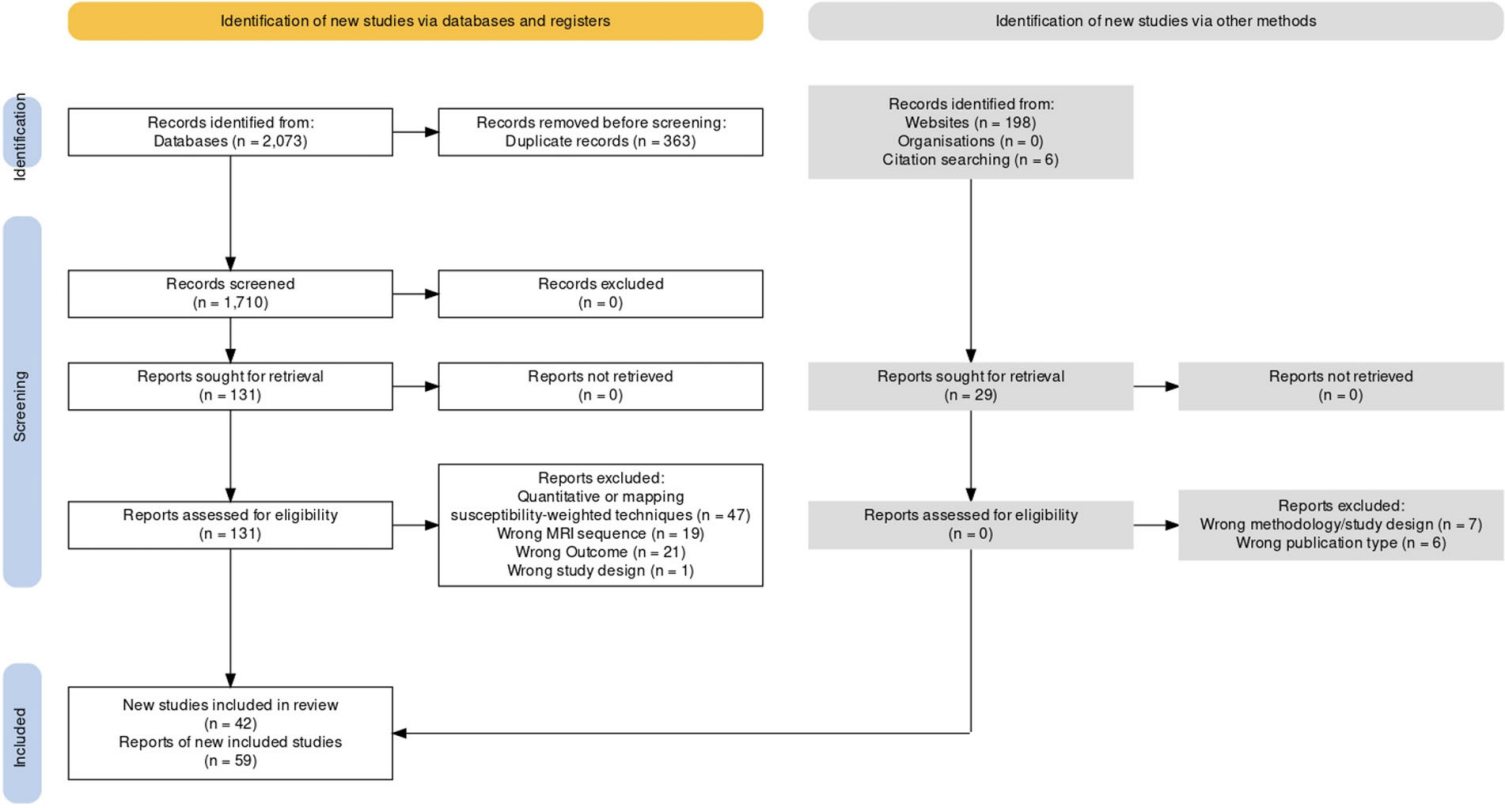
PRISMA flow chart.

**Figure 3:**
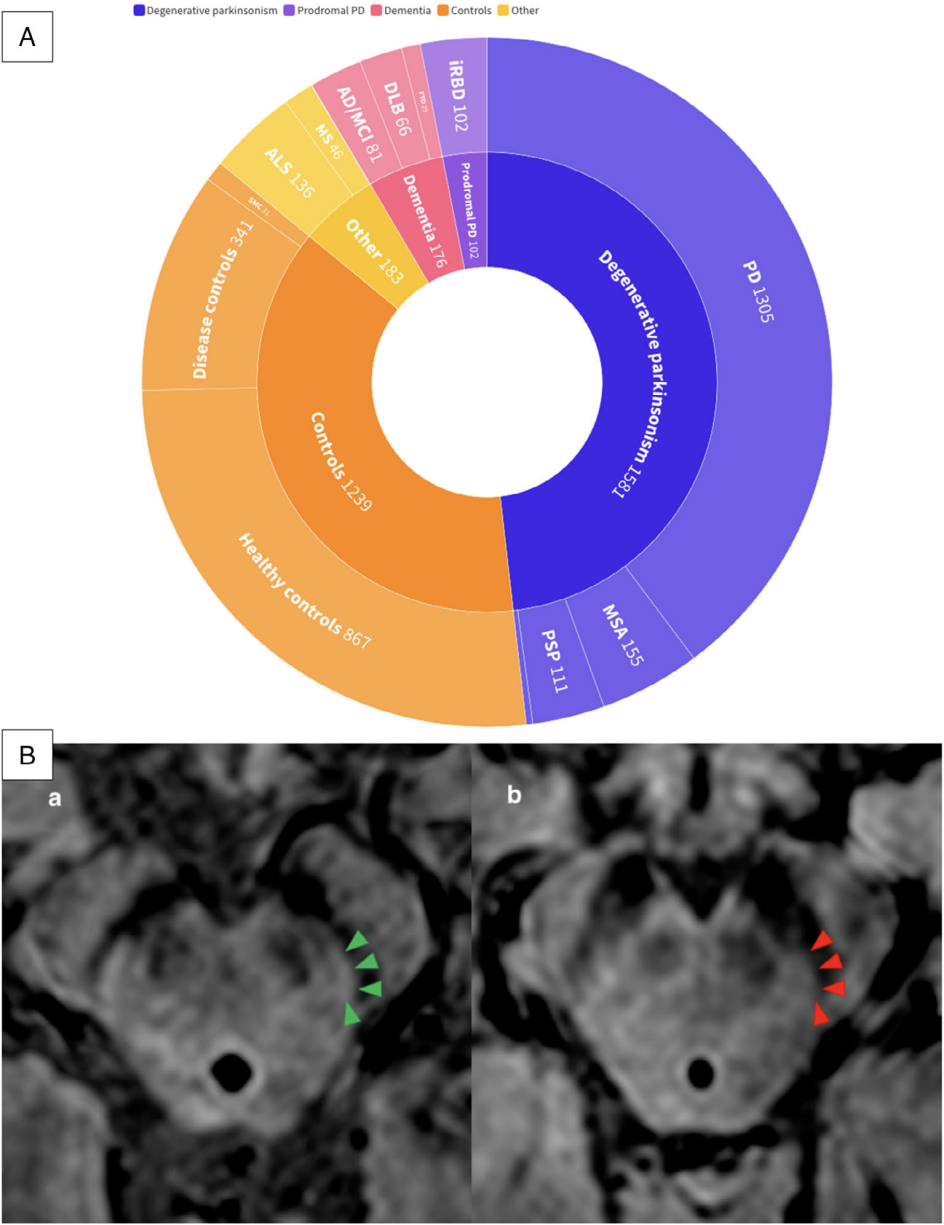
(A) Sunburst chart of the eligible studies’ population (interactive version: https://public.flourish.studio/visualisation/15954619/). (B) Swallow tail sign (STS) evaluation in high‐resolution MRI. STS presence (a) and absence (b). Reproduced with permission from Michler et al.^31^

After deduplication, we retrieved 1710 records from our search on electronic databases (PubMed/MEDLINE, WOS, SCOPUS) and 204 records from possible sources of gray literature (ProQuest, Google Scholar, hand searching, citation searching). At the completion of the screening process, 42 studies (59 reports), with a total number of 3281 patients, were included.^5^, ^6^, ^8^, ^9^, ^10^, ^12^, ^19^, ^20^, ^21^, ^22^, ^23^, ^24^, ^25^, ^26^, ^27^, ^28^, ^29^, ^30^, ^31^, ^32^, ^33^, ^34^, ^35^, ^36^, ^37^, ^38^, ^39^, ^40^, ^41^, ^42^, ^43^, ^44^, ^45^, ^46^, ^47^, ^48^, ^49^, ^50^, ^51^, ^52^, ^53^, ^54^ A PRISMA flow chart is presented in Figure 2. Degenerative parkinsonism subjects comprised the majority of the studies’ population (n = 1581), followed by healthy or disease controls (n = 1239), subjects with other pathologies, like multiple sclerosis and amyotrophic lateral sclerosis (n = 183), subjects with dementia (n = 176) and subjects with prodromal PD. In Figure 3A an interactive sunburst chart of the eligible studies’ population is presented.

In general, there was low risk of bias and low applicability concerns. Patients were mostly recruited consecutively, but due to the retrospective design in most diagnostic accuracy studies, a case–control design was unavoidable. Raters had adequate experience and were reportedly blinded in the vast majority of the studies. Accurate reference standards were used and reported. STS visual assessment and categorization (index test) was sufficiently explained. Our extensive quality assessment according to different study types is presented as Supplementary Material (S2).

We categorized eligible studies into six groups that reflect distinct situations of the evaluation of STS appearance in clinical practice: (1) Differentiation of PD/APS from healthy controls, disease controls and non‐PD/APS movement disorders; (2) Differentiation between PD and APS; (3) Differential diagnosis of Dementia with Lewy Bodies (DLB) from other forms of dementia; (4) STS in prodromal PD; (5) STS in other non‐parkinsonian neurological disorders; (6) The STS in healthy subjects. Even though DLB as a synucleinopathy belongs to APS, we opted to separately present DLB‐focused studies, since they examined STS loss as a biomarker for differentiation from other dementias.

#### Differentiation of PD/APS (excl. DLB) from Healthy Controls, Disease Controls and Non‐PD/APS Movement Disorders

Twenty‐eight studies^5^, ^6^, ^8^, ^12^, ^19^, ^20^, ^21^, ^22^, ^23^, ^24^, ^25^, ^26^, ^27^, ^28^, ^29^, ^30^, ^31^, ^32^, ^33^, ^34^, ^35^, ^36^, ^37^, ^38^, ^39^, ^40^, ^41^, ^42^ examined the diagnostic utility of the STS visualization in discriminating patients with either PD or APS [Multiple System Atrophy (MSA), Progressive Supranuclear Palsy (PSP), Corticobasal Degeneration (CBD)] from healthy controls (HCs), disease controls (DCs) and non‐degenerative (non‐PD/APS) movement disorders. DCs comprised a group of patients with miscellaneous clinical entities including non‐lewy body dementia, cerebrovascular disease, brain tumors, multiple sclerosis (MS), encephalitis, myopathies, normal pressure hydrocephalus (NPH), peripheral vertigo, delirium, encephalitis, mild cognitive impairment (MCI), neuralgia, psychogenic pain und unspecified conditions. The non‐PD/APS movement disorders included essential tremor (ET), vascular parkinsonism (VaP), drug‐induced parkinsonism (DIP), dystonia and dystonic tremor (DysTr), as well as psychogenic tremor. In all studies STS was assessed by two blinded investigators, using mainly 3 T SWI sequences, while two studies used SWI at both 3 T and 1.5 T MRI. Notably, in five studies MRI scanners with a field strength of 7 T were used. The overall inter‐rater agreement calculated with Cohen's kappa ranged from 0.437 to 1. A synopsis is presented in Table S3. In Figure 3B STS absence on axial plane midbrain SWI is presented.

In most studies, abnormal STS appearance on MRI (on either of two sides of SNc) proved to be a relatively trustworthy diagnostic tool with an overall sensitivity of 73–100%, specificity of 75–100%, and diagnostic accuracy of 77–99% for the differentiation of PD and APS patients from patients with non‐degenerative (non‐PD/APS) movement disorder, HCs or DCs. Nevertheless, in a recent retrospective study by Bae et al that included a disease control group in the non‐PD/APS group, the sensitivity and diagnostic accuracy were rather low, 55% and 62%, respectively.^28^ In the study by Prasuhn et al no significant differences regarding the absence of the STS were demonstrated on 3 T SWI between PD patients and matched HCs (sensitivity 37%, specificity 78%).^30^ A high rate of false negative and false positive results was stated, as well as poor ROC‐AUCs. The high rate of false negatives on SWI was also demonstrated in the study by Hernadi et al, in which sensitivity was as low as 49.2%. However, this was compensated by a higher specificity (100%).^42^ Meijer et al reported their second rater's disappointing results (sensitivity 48.2%, specificity 45.4%) with poor inter‐rater agreement (*κ* = 0.35) (first rater: sensitivity 71.2%, specificity 88.99%). Regarding the agreement of asymmetry in the STS assessment with the clinically predominant side, Kathuria et al did not confirm the lateralizing value of STS abnormalities.^32^

Several studies also investigated the level of agreement between STS assessment on SWI and nuclear medicine scans (6‐[18F] FDOPA PET or 123I‐FP‐CIT (DaTscan)) SPECT. Bae et al found a concordance rate of 86.2% between 123I‐FP‐CIT SPECT and abnormal STS at 3 T SWI in a population of 210 individuals (126 PD, 22 APS, 26 HCs and 36 DCs).^22^ Michler et al found an overall good intermodality agreement between 6‐[18F] FDOPA PET and STS evaluation (80%) as well, highlighting however the superiority of 6‐[18F] FDOPA PET.^31^ Haller et al observed a concordance rate of 77% between SWI STS investigation and DaTSCAN SPECT, with DaTscan outperforming SWI, but presenting a considerable diagnostic accuracy improvement after using STS assessment for patient preselection.^33^ The retrospective study by Bae et al concluded to a relatively low level of agreement between SWI and SPECT.^28^ In the study by Kim et al SWI results of abnormal STS were incompatible with DaTSCAN in 4/7 subjects.^37^ Lee et al found a moderate diagnostic agreement between FP‐CIT PET and high resolution‐SWI (HR‐SWI), concerning mostly cases of early‐stage PD.^27^ On the other hand, abnormal F18 DOPA PET was reported in 82/86 PD patients in the study by Kathuria et al, whereas abnormal SWI STS visualization was found in 80/86, indicating high diagnostic agreement between the two methods.^32^

STS loss has been studied in the differentiation of PD from specific non‐degenerative movement disorders as well, like ET and VaP. Akly et al found that up to 4 out of 16 ET patients exhibited abnormal findings on SWI MRI scans and despite false positives, the study disclosed a satisfying sensitivity (93%), specificity (75–88%) and diagnostic accuracy (84–91%).^25^ Zhao et al evaluated STS using a 3 T MRI in 38 HCs, 33 patients with PD and 34 patients with suspected VaP (16).^26^ The authors stated a loss of STS in 31/33 PD and 15/34 VaP patients, pointing towards high sensitivity but a rather low specificity.

#### Differentiation between PD and APS

Several studies focused on examining SWI STS evaluation to differentiate between PD and APS (Table S3). Meijer et al only found marginal diagnostic capacity of STS evaluation to discriminate PD from APS (AUCs between 0.56 and 0.68) in a population of 39 PD, 20 APS (13 MSA‐P, 3 PSP, 1 CBD, 3 DLB, 1 VaP) and 1 VaP patient.^50^ Similarly, Oustwani et al found no difference in the appearance of STS in a population of 25 PD, 11 MSA and 10 PSP/CBD (tauopathy) patients.^8^ Calloni et al showed a profoundly poor specificity (0%) in discriminating 56 PD patients from 30 APS patients (3 MSA‐C, 9 MSA‐P and 18 PSP) at 1.5 or 3 T SWI sequences.^24^ Sugiyama et al reported no significant prevalence of unilateral STS absence among parkinsonian syndromes, including 36 PD, 8 PSP, and 12 MSA (7 MSA‐C and 5 MSA‐P) patients, with the exception of MSA‐C (abnormal results in 3/7 patients).^23^ On the contrary, Wang et al reported 83.3% sensitivity and 89.7% specificity in the delineation of 18 PD from 39 MSA patients (21 MSA‐C and 18 MSA‐P), with sensitivity rising up to 97.4% when combining STS loss with putaminal hypointensity at 3 Tesla SWI.^51^ Kathuria et al observed an abnormal STS appearance in all 14 APS patients included in the study (2 MSA, 12 PSP) compared to 76/86 PD patients.^32^

#### Differential Diagnosis of Dementia with Lewy Bodies (DLB) from Other Forms of Dementia

The diagnostic value of STS loss in the differentiation of DLB from other dementias has been examined in three studies. Firstly, Haller et al observed that STS was absent in a DLB patient, while two patients with a different dementia, AD and FTD, had a normal appearing STS.^43^ Shams et al investigated the appearance of STS using both 1.5 T and 3 T scans in a retrospective multicenter cross‐sectional study with a population of 97 patients (19 DLB, 21 HCs, 20 Frontotemporal dementia (FTD)), 20 AD and 17 with mild cognitive impairment (MCI) reporting a consensus overall sensitivity, specificity and diagnostic accuracy of 63%, 79% and 76%, respectively.^45^ Subsequently Rizzo et al reported even higher measures of diagnostic accuracy (sensitivity, specificity and diagnostic accuracy of 80%, 76% and 77%, respectively) in a similarly designed retrospective study of a smaller population (15 DLB, 11 AD, 8 FTD, 10 HCs) using a field strength of 3 T.^46^ The study by Kamagata et al was also conducted using 3 T MRI scanners and had the highest measures of diagnostic accuracy (sensitivity, specificity and diagnostic accuracy of 93%, 87% and 90%, respectively) in a population of 86 subjects (29 DLB, 18 AD, 13 MCI, 26 HCs).^44^ The authors compared the results of SWI assessment to DAT‐SPECT, in which lower values for specificity and diagnostic accuracy, namely 84% and 88%, were documented. The findings of all four studies are summarized in Table 1.

**TABLE 1:** Synopsis of studies regarding STS absence in Dementia with Lewy Bodies and idiopathic REM sleep behavior disorder

Study	MRI field strength, sequence and section thickness	Disease group (n)	Disease duration in years, mean	Disease severity, mean	Comparator group (n)	Findings	Reliability
Haller et al, 2015	3 T; Venbold SWI; 2 mm	DLB, n = 1	NR	‐	2 AD, n = 1 FTD, n = 1	Nigrosome‐1 hyperintensity was not detected in DLB, in contrast to AD and FTD.	‐
Kamagata et al, 2016	3 T; SWI; 2 mm	DLB, n = 28	2.68	‐	57 AD, n = 18 aMCI, n = 13 HCs, n = 26	DLB vs other dementias/HCs: SE: 93%; SP: 87%; DAC: 90%	0.84
Shams et al, 2017	1.5 T (n = 46) 3 T (n = 51); SWI; 1.5–1.6 mm (n = 36) 2 mm (n = 40)	DLB, n = 19	NR	‐	78 AD, n = 20 FTD, n = 20 MCI, n = 17 SMC, n = 21	DLB vs other dementias/HCs SE: 63%; SP: 79%; DAC: 76%	0.4
Rizzo et al, 2019	3 T; SWI; 1.6 mm	DLB, n = 15	5	UPDRS III: 27	29 AD, n = 11 FTD, n = 8 SMC, n = 10	DLB vs other dementias/HCs: SE: 80%; SP: 76%; DAC: 77%	0.71
De Marzi et al, 2016	3 T; SWI	iRBD, n = 15	5.34	UPDRS III: 1.93	146; PD, n = 104 HCs, n = 42	STS (−): 10/15 (66.6%) of iRBD pts, significantly different from HCs (8/42, 19%), but not from PD patients (83/104, 79.8%). (analysis reported was performed with a conservative approach for non‐diagnostic, low‐quality scans)	*k*= 0.83–1.000
Bae et al, 2017	3 T; single‐ and multiecho SWI; 2 mm	iRBD, n = 18	5.9	UPDRS III: 0.71	36; PD, n = 18 HCs, n = 18	STS (−): 11/18 (61.1%) iRBD pts. 123I‐FP‐ CIT uptake ratios were significantly lower than in HCs and significantly higher than in PD pts. 5/11 STS (−) iRBD pts presented parkinsonism or dementia 18 months after brain MRI. STS (+): 7/18 (38.9%) iRBD pts. 123I‐FP‐CIT results were comparable to HCs.	*k* = 0.944–0.981
Barber et al, 2020	3 T; SWI; 2 mm	iRBD, n = 40	2.5	UPDRS III: 5.6	59 PD, n = 25 HCs, n = 26	STS (−): 11/40 (27.5%) iRBD pts, proportion significantly higher than HCs (2/26, 7.7%) and significantly lower than PD patients (24/25, 96%) iRBD patients with STS (−): higher probability for prodromal PD and significantly lower putamen dopaminergic activity in SPECT/CT. Abnormal DaT SPECT/CT in 14 iRBD patients, of which only 8 (57%) had absence of STS.	Concordance rate: 93%
Zhang et al, 2021	3 T; SWI; 1.6 mm	iRBD, n = 29	6.1	UPDRS III: 4.4	HCs, n = 28	STS (−): 12/29 (41.4%) iRBD pts, proportion significantly higher than HCs (2/28, 7.1%)	ICC = 0.87

AD, Alzheimer disease; a‐MCI, amnesic mild cognitive impairment; DAC, Diagnostic Accuracy; DaT‐SPECT, Dopamine transporter SPECT; DLB, dementia with Lewy bodies; FTD, frontotemporal dementia; HCs, Health Controls; ICC, Intraclass Correlation Coefficient; iRBD, idiopathic REM sleep behavior disorder; *k*, Cohen's kappa; NR, not reported; PD, Parkinson's disease; pts, patients; SE, Sensitivity; SMC, subjective memory complaint; SP, Specificity; SPECT, Single‐photon emission computed tomography; STS, swallow‐tail sign; SWI, susceptibility‐weighted imaging; T, Tesla; UPDRS III, Unified Parkinson's Disease Rating Scale.

#### STS in Prodromal PD

Regarding the STS appearance in possible prodromal stages of PD, De Marzi et al assessed STS loss on 3 T SWI MRI in 15 subjects with idiopathic rapid eye movement sleep behavior disorder (iRBD) that met the ICSD3 diagnostic criteria, comparing their results to 42 HCs and 104 PD subjects.^47^ An abnormal STS appearance was detected in at least 2/3 iRBD patients, similar with the rate in PD patients and significantly different from HCs, with good inter‐rater agreement. Barber et al reported STS loss approximately in one third of iRBD patients (11/40, 27.5%), with an inter‐rater reliability of 0.93.^29^ The proportion was significantly different from the proportion in HCs (2/26, 7.7%) and PD patients (24/25, 96%). iRBD patients with absent STS had a statistically higher probability for prodromal PD, when the MDS research criteria were applied. DaT SPECT/CT showed abnormalities in 14 iRBD patients, of which only 8 (57%) had STS absence on SWI. Bae et al reported STS loss in 11/18 iRBD patients, with an excellent inter‐observer agreement (Cohen's k between 0.944 and 0.981).^48^ Moreover, the authors stated that STS evaluation on 3 T SWI corresponded to 123I‐FP‐CIT SPECT findings, with STS loss being significantly lower than PD patients, but significantly higher compared to HCs. Finally, the STS was absent in 12/29 iRBD patients and 2/28 healthy controls in a study by Zhang et al, indicating statistical significance. Table 1 presents a summary of the above‐mentioned studies.^49^

#### STS in Other Neurological Disorders

Interestingly, Weber et al investigated the STS using 3 T SWI MRI in 46 MS patients and 23 HCs.^9^ They found STS loss in a significant proportion of MS patients (28/46, 60%) compared to HCs (4/23, 17%). There were no differences in age, disease duration or Expanded Disability Status Scale (EDSS) scores between MS patients with absent or present STS. Moreno‐Gambín et al investigated the neuropathologically confirmed TDP‐43 aggregation in the SN of patients with Amyotrophic Lateral Sclerosis (ALS) by evaluating the STS in a prospectively designed cross‐sectional study.^10^ Reportedly, 30% of ALS patients had an absent STS, which however did not correlate with the clinical laterality. STS absence was significantly linked to male sex and poor prognosis. Surprisingly, STS absence was reported by Cui et al in the context of neurosyphilis in a 57‐year‐old Chinese man with progressive cognitive decline, behavioral changes and bilateral hand tremor, initially misdiagnosed as AD.^52^ The authors hypothesized the STS as a new radiological finding in neurosyphilis. Table 2 provides a tabular summary of the afore‐mentioned studies.

**TABLE 2:** Synopsis of studies regarding STS absence in multiple sclerosis, amyotrophic lateral sclerosis, neurosyphilis and parkinsonism‐free individuals

Study	MRI field strength, sequence and section thickness	Disease group (n)	Disease duration in years, mean	Disease severity, mean	Comparator group (n)	Findings	Reliability
Weber et al., 2020	3 T; SWI; 1.5 mm	MS, n = 46	6.5	EDSS: 2	HCs, n = 23	STS loss was found in a significant proportion of MS patients (28/46, 60%) compared to HCs (4/23, 17%). There were no differences in age, disease duration or EDSS scores between MS patients with absent or present STS.	‐
Moreno‐Gambín et al, 2021	3 T; 3D multi‐echo SWI; 2 mm	ALS, n = 136 PMA, n = 16 PLS, n = 22	ALS; 1.7, PMA; 4.1, PLS; 6.3	ALS ALSFRS‐R: 37.37/UMN score: 5.42 PMA ALSFRS‐R: 40.38/UMN score: 0.5 PLS ALSFRS‐R: 33.52/UMN score: 12	‐	30% of ALS patients had an absent STS, however without correlation with clinical laterality. Males and patients with high UMN scores had a significantly higher probability for STS absence (OR; 3.63 and 1.10, respectively). STS absence was associated with poorer prognosis (HR; 1.79)	*k* = 0.69
Cui et al, 2022	SWI	Neurosyphilis, n = 1	5	‐	‐	STS loss on SWI, unaltered after intravenous treatment with Penicillin G.	‐
Schmidt et al, 2017	7 T; SWI; 0.8 mm	‐	‐	‐	HCs, n = 13	Investigation of nigrosome‐1 appearance in healthy individuals with no signs of parkinsonism and a negative family history. The typical STS could be identified in 81% of the participants and was absent in 19%.	ICC = 0.844; alpha = 0.871
Gramsch et al, 2017	7 T; SWI; 1 mm	‐	‐	‐	HCs and DCs, n = 46	The possible association of advancing age (and thus increasing brain iron accumulation) with compromised STS visibility was examined in parkinsonism‐free HCs and DCs. STS appearance was normal in 93%, without evidence of increased iron accumulation in older individuals.	*k* = 0.671–0.777

ALS, amyotrophic lateral sclerosis; ALSFRS‐R, revised ALS functional rating scale; DCs, disease controls; EDSS, Expanded Disability Status Scale; HCs, Health Controls; HR, hazard ratio; ICC, Intraclass Correlation Coefficient; *k*, Cohen's kappa; MS, Multiple sclerosis; NR, not reported; OR, odds ratio; PLS, primary lateral sclerosis; PMA, progressive muscular dystrophy; SPECT, Single‐photon emission computed tomography; STS, swallow‐tail sign; SWI, susceptibility‐weighted imaging; T, Tesla; UMN score, upper motor neuron score.

#### The STS in Healthy Individuals

Schmidt et al investigated STS appearance using 7 T SWI MRI images in 13 healthy subjects with a mean age of 47 years.^53^ Subjects had no clinical signs of parkinsonism and a negative family history for PD. The typical dorsolateral SNc swallow‐tail shape could be identified in 81% of the participants. Thus, the STS was absent in 19% of healthy individuals, with authors suggesting inconsistency due to microstructural organization variabilities of normal STS among healthy individuals.

The study by Gramsch et al, examined the possible association of advancing age with compromised STS visibility due to increasing brain iron accumulation.^54^ The STS of 46 parkinsonism‐free subjects (aged 19–75 years) was investigated on 7 T SWI. Normal STS appearance was found in 93%, with the oldest subjects exhibiting normal findings. The two studies are included in Table 2.

## Conclusion

Swallow tail sign loss and its underlying pathology have been thoroughly studied in the context of nigrosome‐1 susceptibility‐weighted imaging.^55^ Since reliable neuroimaging biomarkers could immensely contribute to both early disease detection and prognostication, STS abnormalities have been explored in a broad spectrum of neurological pathologies. We conducted a systematic review including studies of visual evaluation for STS loss using SWI, excluding quantitative, neuromelanin‐sensitive and diffusion tensor imaging methods. Despite the theoretically more detailed approach of the latter techniques, they are complex and still hardly available in everyday clinical practice. Thus, their review is beyond the scope of the present paper.

For the differentiation of degenerative parkinsonian syndromes from non‐parkinsonian syndromes most studies provided fair to high diagnostic accuracy. However, one should consider contradictory findings, like the poor specificity for neurodegenerative parkinsonism at 1.5T,^41^ the low sensitivity of SWI compared to other MRI techniques^42^ and the disappointingly poor overall diagnostic performance for the differentiation between PD and HCs.^30^, ^50^ Based on our qualitative analysis, we attribute discrepancies to differences in MRI parameters, experimental groups (disease duration/staging), neuroradiologists’ experience and study design. For instance, degenerative parkinsonism groups included APS in some studies, while others only included PD. Comparator groups consisted of HCs, DCs, non‐degenerative movement disorder patients, or were mixed. As also mentioned by Prasuhn et al, we herein underscore the lack of a standardized MRI protocol as a source of heterogeneity,^30^ with MRI field strength and raters’ experience comprising crucial factors for good STS performance. We thus highlight the need for multicenter future studies, with optimized MRI interpretation between different centers.

Regarding the agreement between nuclear scans and MRI, Michler et al, reported higher diagnostic value of 6‐[18F] FDOPA PET compared to STS evaluation on SWI,^31^ with the authors suggesting the recent symptom onset as a possible contributor to the inconsistency between the two methods. In these cases, STS evaluation seems to produce a relatively high rate of false negative results.^27^, ^42^ This suggestion should admittedly be of concern, since diagnostic confusion in clinical practice mostly concerns the early stages of parkinsonism. Similarly, in studies with reports of good concordance rate between the two methods, nuclear scans outperformed SWI in terms of sensitivity and overall diagnostic accuracy^22^, ^33^; thus, it could be argued that the STS assessment could be of use for the preselected patients, distinguishing possible candidates for nuclear scans in cases of abnormal or ambiguous STS appearance on MRI scans.^33^

The lateralizing value of STS abnormality in relation to the most affected side was questioned in the study by Kathuria et al, in which the mean duration of degenerative parkinsonian syndromes was 3.1 years.^32^ Studies using nuclear scans have demonstrated that the asymmetry between the more and less affected side declines over the course of disease.^56^ Interestingly however, satisfactory contralateral correlation was demonstrated in PD populations with a mean disease duration of 9 months using either 3D multi‐echo data image combination^57^ and 5.2 years using high‐resolution 3D gradient echo.^56^ Thus, other than disease duration could be involved in the correlation of PD asymmetry and laterality in imaging.

Our review results suggest that STS evaluation in differentiating between PD and the majority of APS might be of poor diagnostic value. Although Wang et al. were able to successfully delineate PD patients from MSA patients using STS evaluation,^51^ it should be taken into consideration that most MSA patients had the cerebellar type of the disease, MSA‐C, an entity that has been argued to sometimes present normal nigrostriatal dopaminergic innervation instead of degeneration on nuclear scans.^57^ We note that the normal STS sometimes found in APS patients can be attributed to the fact that dopaminergic neurons are thought to degenerate by a “dying back” mechanism, indicating a larger extent of terminal loss in the striatum than in the SN.^31^ We argue, this explanation could also help interpret the relatively higher rate of disagreement between 6‐[18F] FDOPA PET and STS evaluation in APS, compared to PD, since the first test mirrors presynaptic dopaminergic deficit, while the second comprises a direct depiction of nigrosome‐1 microstructure.

For differentiating DLB from other forms of dementia, data from STS assessment were encouraging,^43^, ^44^, ^45^, ^46^ as DLB is categorized among synucleinopathies and has an underlying pathology similar to PD.^58^ It has been demonstrated that these patients frequently exhibit presynaptic dopaminergic deficits in nuclear scans.^59^ Taking into account the lack of reliable, easy‐to‐perform neuroimaging biomarkers for clinically ambiguous cases, STS assessment could play a significant intermediate role in the correct categorization of patients who would need further workup, but standardized studies are required in order to reach a safe conclusion.

It is known that iRBD can precede the manifestation of synucleinopathies with risk estimates of 17.7% at 5 years and 40.6% at 10 years.^60^ The discovery of neuroimaging biomarkers that could help detect patients with iRBD at high risk for conversion to PD could be the first step in selecting subjects during the prodromal PD state for early intervention with possible neuroprotective agents. We present studies with a satisfactory level of agreement between STS assessment and nuclear scan findings in iRBD, but further research with longitudinal clinical follow‐up is needed in order to confirm the method's utility in risk prediction.

Our results indicate that STS assessment might be of particular clinical utility in miscellaneous neurological disorders. Admittedly, the use of iron‐sensitive MRI techniques in MS has been proved useful in the context of disease progression.^61^ Weber et al.^9^ documented significant differences between MS patients and HCs, regarding the detection of STS loss using 3 T SWI. Their results are in line with the 2014 study by Blazejewska et al,^62^ in which MS patients presented significantly increased magnetic susceptibility within the SN and the red nucleus, consistent with iron deposition, using T_2_*‐weighted ultra‐high‐field 7 T susceptibility maps. Interestingly, in both studies no correlation of magnetic susceptibility with disease duration or EDSS scores of MS patients was found. Further research on susceptibility changes of the SN in specific MS subgroups (eg, clinically isolated syndrome) might establish the method's clinical utility in disease monitoring and give insights or even guide studies on MS pathophysiology. Regarding ALS, Moreno‐Gambín et al have correlated STS loss with higher upper motor neuron scores and poorer prognosis.^10^ Their results are consistent with previously shown SNc impairment due to TDP‐43 aggregation.^63^ Hence, despite the need for neuropathological confirmation, SWI STS loss may comprise a prognostication biomarker in ALS.

The results of eligible studies on the consistency of its appearance in healthy participants seem to be debatable, with authors hypothesizing individual anatomic variants in the midbrain.^53^ Indeed, midbrain microvessels may cause STS appearance differences in aged individuals without parkinsonism, as confirmed by the combination of 7 T MRI and post‐mortem histology with iron and myelin staining.^63^ Thus, SWI hypointensity is thought to result from local susceptibility changes, attributed to iron deposits and to vascular structures containing deoxygenated blood.^63^ This finding could explain the false positive results concerning HCs, especially if less experienced evaluators are involved.

STS evaluation through visual inspection could be an easily applicable neuroimaging biomarker in everyday clinical practice. However, despite reports on its robustness regardless of the rater's experience,^12^ the methodology undeniably has important limitations, associated with its inherent subjectivity. Firstly, even though the definitions for unilateral and bilateral STS loss in the included studies were similar or almost identical, frequent variations of the normal STS appearance were illustrated (eg, “loop sign”),^12^ that should be taken into account by raters. Additionally, Cosottini et al have evaluated the STS at different midbrain levels, whereas in the majority of studies, no such detail existed.^20^ A recent study also highlighted the need for expert neuroradiologists for correct STS categorization.^38^ Understandably, normal variations, the evaluated midbrain levels and raters’ experience should be taken into consideration for STS utility in clinical practice.

Advanced quantitative techniques, like the estimation of free water, QSM and NM‐MRI, could have even more reproducible and objective results through the use of standardized methodologies.^64^, ^65^, ^66^ Interestingly, the detection of STS loss and its size have also been studied using deep‐learning algorithms, with impressive diagnostic accuracy in discriminating PD patients (including early‐stage) from HCs. Nevertheless, these techniques are available mostly for research purposes in sparse Radiology departments, rendering their use limited and widely isolated from everyday clinical routine, at least for the time being.

The loss of the swallow tail sign on SWI has shown significant diagnostic potential, mainly for the differentiation of degenerative parkinsonian from non‐degenerative parkinsonian movement disorders. It remains a promising biomarker for the discrimination of DLB from other dementias and for the detection of iRBD patients, who are at high risk for conversion to PD. We suggest a possible intermediate role of the STS assessment in the diagnostic workup of the above‐mentioned pathologies and encourage the future research of STS visualization using standardized MRI protocols and optimized guidelines for interpretation of results.

## Author Roles

(1) Research project: A. Conception, B. Organization, C. Execution; (2) Data synthesis: A. Data extraction, B. Execution, C. Review, and Critique; (3) Manuscript Preparation: A. Writing of the first draft, B. Review, and Critique.

V.S.T.: 1B, 1C, 2A, 2B, 2C, 3A, 3B.

K.E.: 1A, 1B, 1C, 2A, 1B, 3A.

T.M.: 2B, 2C, 3B.

G.K.: 1C, 2A, 2B.

B.F.: 1A, 1B, 2C, 3B.

M.A.: 1A, 1B, 2C, 3B.

## Disclosures

**Ethical Compliance Statement:** The authors confirm that neither the approval of an institutional review board or patient consent were required for this work. We confirm that we have read the Journal's position on issues involved in ethical publication and affirm that this work is consistent with those guidelines.

**Funding Sources and Conflict of Interest:** The publication of the article in OA mode was financially supported by HEAL‐Link. The authors declare that there are no conflicts of interest relevant to this work.

**Financial Disclosures for the Previous 12 Months:** The authors declare that there are no additional disclosures to report.

## Supporting information


**Data S1.** PRISMA checklist.

**Data S2.** Quality assessment.

**TABLE S1.** The complete search strategy for MEDLINE (October 26, 2023).

**TABLE S2.** The 20 keywords with the highest weight (occurrence rate) along with their total link strength.

**TABLE S3.** Synopsis of studies that included subjects with degenerative parkinsonian syndromes (PD/APS) with or without comparator groups (HCs, DCs, or both). Study characteristics and measures of diagnostic value of an absent STS on either side of SNc are presented.

## Data Availability

The full search strategy for MEDLINE is presented in our Supplementary Material.
