# Supplementary material for: Is the Swallow Tail Sign a Useful Imaging Biomarker in Clinical Neurology? A Systematic Review
Source: Mov Disord Clin Pract. 2024 Dec 17;12(2):134–47. doi: 10.1002/mdc3.14304 (PMC11802665; doi:10.1002/mdc3.14304)
Supplement: Supplementary file 2 — Data S2. Quality assessment. [file MDC3-12-134-s002.docx]

|  | Haller et al., 2016 | | | | | | Cui et al., 2022 | | | | | |
| --- | --- | --- | --- | --- | --- | --- | --- | --- | --- | --- | --- | --- |
|  | Yes | No | | Unclear | | Not applicable | Yes | No | | Unclear | | Not applicable |
| Were patient’s demographic characteristics clearly  described? | X |  | |  | |  | X |  | |  | |  |
| Was the patient’s history clearly described and presented  as a timeline? |  |  | | X | |  | X |  | |  | |  |
| Was the current clinical condition of the patient on  presentation clearly described? |  |  | | X | |  | X |  | |  | |  |
| Were diagnostic tests or assessment methods and the  results clearly described? | X |  | |  | |  | X |  | |  | |  |
| Was the intervention(s) or treatment procedure(s) clearly  described? |  |  | |  | | X | X |  | |  | |  |
| Was the post-intervention clinical condition clearly  described? |  |  | |  | | X | X |  | |  | |  |
| Were adverse events (harms) or unanticipated events  identified and described? |  |  | |  | | X | X |  | |  | |  |
| Does the case report provide takeaway lessons? | X |  | |  | |  | X |  | |  | |  |
|  | Include | | Exclude | | Seek further info | | Include | | Exclude | | Seek further info | |
| Overall appraisal: | X | |  | |  | | X | |  | |  | |

**TITLE: Nigrosome-1 Visualization on SWI: a Systematic Review of the Swallow Tail Sign in Clinical Neurology**

**JBI CRITICAL APPRAISAL CHECKLIST FOR CASE REPORTS**

Reviewers: Vasilis-Spyridon Tseriotis; Theodoros Mavridis

|  | Schmidt et al., 2017 | | | | | | Gramsch et al., 2017 | | | | | |
| --- | --- | --- | --- | --- | --- | --- | --- | --- | --- | --- | --- | --- |
|  | Yes | No | | Unclear | | Not applicable | Yes | No | | Unclear | | Not applicable |
| Was the sample frame appropriate to address the target population? | X |  | |  | |  | X |  | |  | |  |
| Were study participants sampled in an appropriate way? | X |  | |  | |  | X |  | |  | |  |
| Was the sample size adequate? |  |  | | X | |  | X |  | |  | |  |
| Were the study subjects and the setting described in detail? | X |  | |  | |  | X |  | |  | |  |
| Was the data analysis conducted with sufficient coverage of the identified sample? | X |  | |  | |  | X |  | |  | |  |
| Were valid methods used for the identification of the condition? | X |  | |  | | X | X |  | |  | |  |
| Was the condition measured in a standard, reliable way for all participants? | X |  | |  | | X | X |  | |  | |  |
| Was there appropriate statistical analysis? | X |  | |  | |  | X |  | |  | |  |
| Was the response rate adequate, and if not, was the low response rate managed appropriately? |  |  | |  | | X |  |  | |  | | X |
|  | Include | | Exclude | | Seek further info | | Include | | Exclude | | Seek further info | |
| Overall appraisal: | X | |  | |  | | X | |  | |  | |

**JBI CRITICAL APPRAISAL CHECKLIST FOR STUDIES REPORTING PREVALENCE DATA**

Reviewers: Vasilis-Spyridon Tseriotis; Theodoros Mavridis

**QUADAS-2 TOOL FOR THE QUALITY ASSESSMENT OF DIAGNOSTIC ACCURACY STUDIES**

Reviewers: Vasilis-Spyridon Tseriotis; Theodoros Mavridis


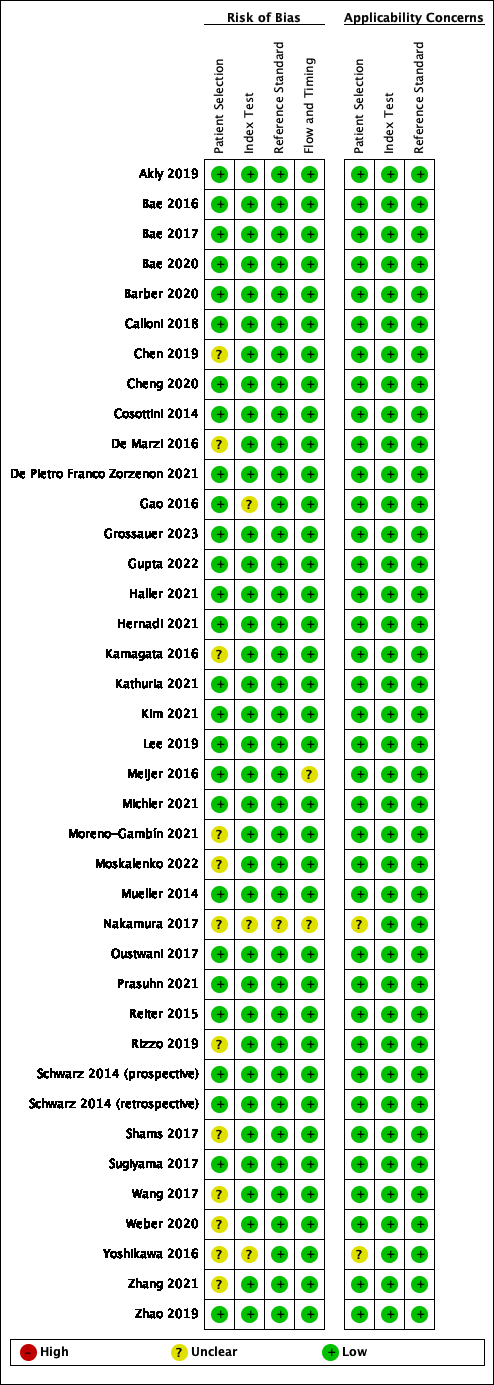


**Figure 1:** Risk of bias and applicability concerns summary: review authors' judgements about each domain for each included study


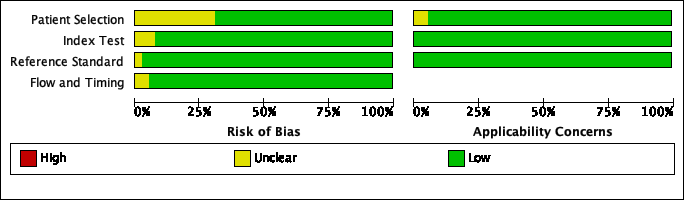


**Figure 2:** Risk of bias and applicability concerns graph: review authors' judgements about each domain presented as percentages across included studies
