# Supplementary material for: Is the Swallow Tail Sign a Useful Imaging Biomarker in Clinical Neurology? A Systematic Review
Source: Mov Disord Clin Pract. 2024 Dec 17;12(2):134–47. doi: 10.1002/mdc3.14304 (PMC11802665; doi:10.1002/mdc3.14304)
Supplement: Supplementary file 3 — TABLE S1. The complete search strategy for MEDLINE (October 26, 2023). [file MDC3-12-134-s004.docx]

**Table S1:** The complete search strategy for MEDLINE (October 26^th^, 2023)

| Search | Query | Items found |
| --- | --- | --- |
| #1 | Search: **"swallow tail sign" OR "swallow-tail sign" OR "swallow tail appearance" OR "dorsolateral nigral hyperintensity" OR "nigral hyperintensity" OR "dorsolateral substantia nigra" OR "nigrosome-1" OR "nigrosome 1" OR "substantia nigra"** | 30,583 |
| #2 | Search: **"susceptibility-weighted imaging" OR "susceptibility weighted imaging" OR SWI OR "magnetic resonance imaging" OR MRI OR "Magnetic Resonance Imaging"[Mesh]** | 761,434 |
| #3 | Search: **#1 AND #2** | 2,252 |
| #4 | Search: **#1 AND #2** Filters: from 2010 - 2023 | 1601 |
