# Supplementary material for: Is the Swallow Tail Sign a Useful Imaging Biomarker in Clinical Neurology? A Systematic Review
Source: Mov Disord Clin Pract. 2024 Dec 17;12(2):134–47. doi: 10.1002/mdc3.14304 (PMC11802665; doi:10.1002/mdc3.14304)
Supplement: Supplementary file 4 — TABLE S2. The 20 keywords with the highest weight (occurrence rate) along with their total link strength. [file MDC3-12-134-s005.docx]

| **Table S2:** The 20 keywords with the highest weight (occurrence rate) along with their total link strength | | |
| --- | --- | --- |
| **Keyword** | **Weight (occurrence)** | **Total link strength** |
| Substantia nigra | 1049 | 7111 |
| Parkinson’s disease | 938 | 6444 |
| MRI | 894 | 6008 |
| Iron | 504 | 3576 |
| Basal ganglia | 321 | 2220 |
| Diagnosis | 251 | 1861 |
| Neurodegeneration | 200 | 1387 |
| Functional MRI | 180 | 1157 |
| Multiple system atrophy | 169 | 1376 |
| Neuromelanin | 169 | 1374 |
| Alzheimer’s disease | 167 | 1234 |
| Dopamine | 153 | 1073 |
| Positron Emission Tomography | 150 | 988 |
| Quantitative susceptibility mapping | 145 | 1069 |
| Progressive supranuclear palsy | 143 | 1115 |
| Locus coeruleus | 134 | 1003 |
| Susceptibility weighted imaging | 132 | 987 |
| In-vivo | 115 | 838 |
| Deep brain stimulation | 104 | 677 |
| Diffusion weighted imaging | 101 | 748 |
