# Supplementary material for: Is the Swallow Tail Sign a Useful Imaging Biomarker in Clinical Neurology? A Systematic Review
Source: Mov Disord Clin Pract. 2024 Dec 17;12(2):134–47. doi: 10.1002/mdc3.14304 (PMC11802665; doi:10.1002/mdc3.14304)
Supplement: Supplementary file 5 — TABLE S3. Synopsis of studies that included subjects with degenerative parkinsonian syndromes (PD/APS) with or without comparator groups (HCs, DCs, or both). Study characteristics and measures of diagnostic value of an absent STS on either side of SNc are presented. [file MDC3-12-134-s001.docx]

| **Table S3:** Synopsis of studies that included subjects with degenerative parkinsonian syndromes (PD/APS) with or without comparator groups (HCs, DCs, or both). Study characteristics and measures of diagnostic value of an absent STS on either side of SNc are presented | | | | | | | | | |
| --- | --- | --- | --- | --- | --- | --- | --- | --- | --- |
| **Study and year of publication** | **MRI field strength, sequence and section thickness** | **PD pts (n)** | **APS pts (n)** | **PD or PD/APS duration in years, mean** | **PD or PD/APS disease severity, mean** | **HCs (n)** | **Other (n)** | **Diagnostic value (SE/SP/DAC)** | **Inter-rater reliability** |
| Mueller et al., 2014 | 3T; SWI | 35 | - | 8 | NR | 14 | - | PD vs HCs: 85.7%, 100%, 89.8% | - |
| Cosottini et al., 2014 | 7T; 3D multiecho SWI; 1.2 mm | 17 | - | 2.3 | H&Y: 1.7; UPDRS III: 17.8 | 13 | - | PD vs HCs: 100%; 96.2%; 98.4% | k=0.932 |
| Schwarz et al., 2014 (prosp. case-control study) | 3T; High-resolution 3D SWI | 10 | - | 4 | H&Y: 1.85; UPDRS: 32.5 | 9 | - | PD vs HCs: 80%; 89%; 84% | k=0.82 |
| Schwarz et al., 2014 (retr. cross-sectional study) | 3Τ; High-resolution T2*/3D SWI | 9 | - | NR | NR | - | 81 DCs [dementia, cerebrovascular disease, brain tumors, multiple sclerosis, encephalitis] | PD vs DCs: 100%; 95%; 96% | k=0.82 |
| Reiter et al., 2015 | 3T; SWI; 2.4 mm | 90 | 42 [MSA, n=21; PSP, n=21] | 4.6 | H&Y: 2.58; UPDRS III: 31.4 | 35 | - | PD/APS vs HCs: 94.7%; 97.2%; 95.2% | k=0.838 |
| Gao et al., 2016 | 3T; SWI; 2 mm | 54 | - | 7.72 | NR | - | 51 non-PD [mild brain atrophy, dif- ferent degrees of cerebral infarction, cerebral hemorrhage, tumors] | PD vs non-PD: 100%, 96.1%, 98.1% | NR |
| Yoshikawa et al., 2016 | 3T; SWI; 2 mm | 7 | 5 [MSA-P, n=2  PSP, n=3] | NR | NR | 10 | - | PD/APS vs HCs: 92%; 100% | NR |
| Bae et al., 2016 | 3T; Single- and multi-echo FFE-SWI | 126 | 22 [MSA, n=11; PSP, n=11] | 2.24 | H&Y: 1.96; UPDRS III: 20.50 | 26 | 36 non-PD/APS movement disorders [DIP, n=6; DysTr, n=2; ET, n=8; Psych. Disorder, n=1; VaP, n=19] | PD/APS vs HCs: 88.8%; 83.6%; 87.1% | k=0.83 |
| Meijer et al., 2016 | 3T; 3D gradient echo SWI; 3mm | 39 in total* | 21 [MSA-P, n=13; PSP, n=3; DLB, n=3; VaP, n=; CBS, n=1] | PD; 1.8, APS; 2.37 | PD; H&Y: 1.7/UPDRS III: 32.4 APS; H&Y: 2.4/UPDRS III: 45.7 | 12 | - | PD/APS vs HCs R1: 71.2%; 88.9%; 73.8%, R2: 48.2%; 45.4%; 47.8% PD vs APS R1: 71.9%; 70%; 71.2%, R2: 37.2%; 33.3%; 35.7% | k=0.35 |
| Oustwani et al., 2017 | 1.5/3T; SWI; 1.6 - 2.25 mm | 25 | 21 [MSA, n=11; PSP/CBD, n=10] | NR | NR | 14 | - | PD/APS vs HCs: 85%; 79%; 83% PD vs APS: 76%; 4.76%; 43.5% | k=0.83-1 |
| Nakamura et al., 2017 | 3T; SWI | 9 | 7 | NR | - | 32 | - | PD vs HCs: 77.8%; 84.8%; 82.9% | NR |
| Sugiyama et al., 2017 | 3T; SWI-like images reconstructed from PADRE; 1 mm | 36 | 20 [MSA, n=12; PSP, n=8] | 7.11 | H&Y: 3.36 | 33 | - | PD/APS vs HCs: 98%; 93.9%; 96.3% | k=0.74 |
| Wang et al., 2017 | 3T; SWI (3D-FLASH T2WI sequence); 0.8 mm | 18 | 39 [MSA-C, n=21; MSA-P, n=18] | PD: 7.67; APS: 3.96 | PD; H&Y: 2.72; APS; H&Y: 2.87 | 31 | - | PD vs MSA: 83.3.%; 89.7%; 87.2% PD vs HCs: 83.3%; 100%; 93.9% PD/APS vs HCs: 33.3%; 100%; 56.8% | k=0.72 |
| Calloni et al., 2018 | 3T; SWI; 1.4 mm | 56 | 30 [MSA-C, n=3; MSA-P, n=9; PSP, n=18] | 8.26 | H%Y: 2.1 | 24 | 16 DCs [VaP, ET, myopathies, NPH, peripheral vertigo, dementia (non-DLB)] | PD/APS vs HCs/DCs: 97.7%; 85%; 93.7% PD vs APS: 96.43%; 0%, 62.8% | - |
| Akly et al., 2019 | 3T; 3D SWI | 16 | - | 7.1 | H&Y: 1.94; UPDRS III: 22.1 | - | ET, n=16 | PD vs ET: 93%; 81.5%; 87.5% | k=0.87 |
| Chen et al., 2019 | 3T; SWI | 32 | - | STS (-): 4.6 STS (+): 1.5 | STS (-) H&Y: 2, UPDRS III: 24 STS (+) H&Y: 1.5, UPDRS III: 13 | 20 | - | 26/32 PD pts were STS (-) | NR |
| Zhao et al., 2019 | 3T; SWI; 1.2 mm | 33 | - | 4.7 | H&Y: 1-2 (n=16); H&Y: 1-3 (n=17) | 38 | VaP, n=34 | PD vs HCs/VaP: 93.9%; 92.1%; 91.5% | k=0.87; k=0.93 |
| Lee et al., 2020 | 3T; High-resolution SWI; 1 mm | 31 | 3 (MSA) | 2 | H&Y: 1.5; UPDRS III: 20.3 | 21 | 14 DCs [vascular dementia, VaP, myoclonus, ET, DIP, dystonia, psych. tremor, delirium, encephalitis] | PD/APS vs HCs/DCs: 73.5%; 80%; 76.8% PD/APS vs DCs: 73.5%; 92.9%; 79.2% | k=0.8 |
| Cheng et al., 2020 | 3T; SWI; 1.34 mm | 57 | 14 | NR | NR | 80 | ET, n=9 | PD/APS vs HCs: 78.8%; 97.5%; 88.7% | α=0.82 |
| Bae et al., 2020 | 3T; SWI; 1 mm | 98 | - | H%Y: 2; UPDRS III: 17.7 | H&Y: 1-3 | - | 27 non-PD/APS movement disorders [VaP, DIP, ET] | PD vs DCs: 55.1%; 88.9%; 62.4% | k=0.908 |
| Barber et al., 2020 | 3T; SWI; | 25 | - | 5.2 | UPDRS III: 31.9 | 32 | iRBD, n=46 (results concerning STS in iRBD are presented in Table 4) | PD vs HCs: 96%, 92%; 94.1% | k=0.930 |
| Hernadi et al., 2021 | 3T; SWI; 1.5 mm | 14 | - | 3.7 | H&Y: 1.55; UPDRS III: 8.3 | 19 | - | PD vs HCs: 42.9%, 100% |  |
| Prasuhn et al., 2021 | 3T; SWI | 44 | - | 7.4 | H&Y: 1.7; UPDRS III: 27.3 | 50 | - | PD vs HCs: 37%; 78% | k=0.67 |
| Michler et al., 2021 | 3T; SWI; 0.5 mm | 18 | 9 [MSA, n=1; PSP, n=6; CBD, n=2] | 2.56 | NR | - | 16 DCs [ET, n=1; VaP, n=3; DIP, n=2; RLS, n=1; Ataxia, n=1; MND, n=1; AD, n=1; HD, n=1] | PD/APS vs DCs: 82%; 75%; 79% | k=0.611 |
| Kathuria et al., 2021 | 3T; VenoBOLD and high-resolution SWI | 86 | 14 [MSA, n=2; PSP, n=12] | 3.1 | H&Y: 2.4; UPDRS III: 75.1 | 15 | - | PD/APS vs HCs: 94%; 80% PD vs HCs: 93%; 80% APS vs HCs: 100%; 80% | - |
| Haller et al., 2021 | 3T; SWI | 31 | 23 [MSA-P, n=1; MSA-C, n=2; LBD, n=2; CBD, n=2 Unspecified APS, n=6; Unspecified parkinsonism, n=10] | NR | NR | 24 | 18 DCs [Tremor, n=7 Dementia, n=3; MCI, n=3; NPH, n=1 Neuralgia, n=1; Psych. pain, n=1; Unspecified, n=2] | PD/APS vs HCs: 74%; 92%; 83% | k=0.437-0.487 |
| Kim et al., 2021 | 3T; SWI; 1 mm | 3 | 4 [Probable MSA, n=3; Probable PSP, n=1] | NR | - | - | 13 DCs [VaP, n=3; Psych. gait disorder, n=1; DIP, n=2; ET, n=1; Cervical dystonia, n=1; Static encephalopathy, n=1; Gait disorder of unknown etiology, n=1; Transient Parkinsonism, n=2; VPA induced parkinsonism, n=1] | STS (+) in 2/3 iPD, 3/3 MSA, 1/3 VaP, 1/1 psych. gait disorder, 2/2 DIP, 1/1 cervical dystonia, 1/1 static encephalopathy, 1/1 gait disorder of unknown etiology | - |
| De Pietro Franco Zorzenon et al., 2021 | 3T; SWI; 2 mm | 33 | - | 8.4 | UPDRS III: 35.85 | 15 | - | PD vs HCs: 91%, 88%, 90% (evaluation by an expert neuroradiologist) | k=0.7 |
| Gupta et al., 2022 | 3T; SWI; 1mm | 45 PD/APS patients – exact numbers NR | | NR | NR | 45 | - | PD/APS vs HCs: 75.5%; 97.7%; 86.6% | k=0.80 |
| Moskalenko et al., 2022 | 3T; SWI; 1.5 mm | 20 | - | NR | NR | NR | ET, n=10 | PD vs ET: 70%, 100%, 80% | NR |
| Grossauer et al., 2023 | 1.5T, 3T; SWI; 2.4 mm | 41 | 55; [MSA, n=22; PSP, n=23] | 4 | H&Y: 2.8; UPDRS III: 32.7 | 33 | - | 3T, PD/APS vs HCs: 95.8%; 95.5%; 95.7% 1.5T, PD/APS vs HCs: 97.2%; 31.8%; 81.7% | 3T; k=0.92 1.5T; k=0.54 |

PD: Parkinson disease; APS: atypical parkinsonian syndromes; MSA: multiple system atrophy; PSP: progressive supranuclear palsy; CBD: corticobasal degeneration; pts: patients; HCs: healthy controls; AD: Alzheimer disease; MCI: mild cognitive impairment; DCs: disease controls; DIP: drug-induced parkinsonism; DysTr: dystonic tremor; ET: essential tremor; VaP: vascular parkinsonism; MS: multiple sclerosis; NPH: normal pressure hydrocephalus; DLB: dementia with Lewy bodies; RLS: restless leg syndrome; MND: motor neuron disease; HD: Huntington disease; VPA: valproic acid; iRBD: idiopathic REM sleep behavior disorder; SWI susceptibility-weighted imaging; PADRE: phase difference enhanced imaging; H&Y: Hoehn and Yahr Scale; UPDRS: Unified Parkinson's Disease Rating Scale; k: Cohen’s kappa for inter-rater reliability; SE: Sensitivity; SP: Specificity; DAC: Diagnostic Accuracy; R1: reader 1; R2: reader 2; NR: not reported
*Indecisive scans: PD; 7 for reader 1 and 4 for reader 2, APS; 1 for reader 1 and 0 for reader 2, HCs; 3 for reader 1 and 1 for reader 2

Notes: Only the numbers of subjects used for comparisons and for calculation of diagnostic value are herein reported, and not the overall study population (e.g., patients with indecisive scans were excluded if they were not accounted in the analysis of the original studies).

In the columns PD/APS disease duration and PD/APS disease severity we report the combined means of both the PD and APS group, calculated by us, except for the studies that included only PD or only APS; in that case the corresponding value was extracted from the study report.
